# Determinants of birth asphyxia among newborns in Debre Berhan referral hospital, Debre Berhan, Ethiopia: a case-control study

**DOI:** 10.1186/s12887-022-03223-3

**Published:** 2022-03-30

**Authors:** Sisay Shine Tegegnework, Yeshfanos Tekola Gebre, Sindew Mahmud Ahmed, Abrham Shitaw Tewachew

**Affiliations:** 1 Asrat Woldeyus Health Science Campus, School of Public Health, Debre Berhan University, Debre Berhan, Ethiopia; 2 Asrat Woldeyus Health Science Campus, School of Nursing and Midwifery, Debre Berhan University, Debre Berhan, Ethiopia; 3 Nursing Department, Minlik II College of Medicine and Health Science, Kotebe Metropolitan University, Addis Ababa, Ethiopia; 4 Asrat Woldeyus Health Science Campus, School of Medicine, Debre Berhan University, Debre Berhan, Ethiopia

**Keywords:** Birth asphyxia, Determinant, Newborn, Debre Berhan, Ethiopia

## Abstract

**Background:**

Birth asphyxia is the major public health problem in the world. It is estimated that around 23% of all newborn deaths are caused by birth asphyxia worldwide. Birth asphyxia is the top three causes of newborn deaths in sub-Saharan Africa and more than one-third of deaths in Ethiopia. Therefore, the aim of this study was to identify determinants of birth asphyxia which can play a crucial role to decrease the death of newborns.

**Methods:**

Unmatched case-control study design was implemented among 276 (92 cases and 184 controls) newborns from January 1st to March 30th, 2020. A systematic sampling technique was used to select the study participants. Data were collected by using a semi-structured interviewer-administered questionnaire and document review by trained nurses and midwives who work at the delivery ward of the hospitals. Bivariate logistic regression analysis was done to identify determinants of birth asphyxia. Adjusted odds ratios with 95% confidence intervals and *p*-value less than *and equal to* 0.05 were used to assess the level of significance.

**Results:**

In this study, maternal education of being can’t read & write [AOR = 4.7, 95% CI: (1.2, 11.9)], ante-partum hemorrhage [AOR = 7.7, 95% CI: (1.5, 18.5)], prolonged labor [AOR =13.5, 95% CI: (2.0, 19.4)], meconium stained amniotic fluid [AOR = 11.3, 95% CI: (2.7, 39.5)], breech fetal presentation [AOR = 4.5, 95% CI: (2.0, 8.4)] and preterm birth [AOR: 4.1, 95% CI: (1.8, 9.2)] were factors which showed significantly associated with birth asphyxia among newborns.

**Conclusions:**

In this study, maternal education can’t read & write, antepartum hemorrhage, prolonged labor, stained amniotic fluid, breech fetal presentation, preterm birth were significantly associated with birth asphyxia. So, educating mothers to enhance health-seeking behaviors and close monitoring of the labor and fetus presentation were recommended to reduce birth asphyxia.

**Supplementary Information:**

The online version contains supplementary material available at 10.1186/s12887-022-03223-3.

## Introduction

Birth asphyxia is defined as the failure of the newborn to initiate and sustain adequate respiration after delivery [1]. *It is also an existence of umbilical cord arterial pH < 7; APGAR score of 0–3 for longer than 5 min; neurological manifestations such as seizures, hypercapnia, metabolic acidosis and hypoxic-ischemic encephalopathy* [2]. It is the major public health problem among under-five children in the world. Globally, 4 million or 23.0% of newborn deaths occur yearly due to birth asphyxia, representing the fifth (38.0%) largest cause of under-5 year’s children deaths [3]. The problem is severe in developing countries at which 120 million newborns develop birth asphyxia and the cause for 900,000 deaths every year. In sub-Saharan African countries 24.0% of newborn deaths were due to birth asphyxia [4].

In Ethiopia, birth asphyxia is the cause of more than one-third of newborn deaths [5]. The effect of birth asphyxia is not limited only to death but also has a short and long-term neurodevelopment squeal including cognitive and motor disabilities which are almost untreatable [6, 7]. In order to alleviate this problem, national newborn training and guidelines are well established for those health professionals who were attending labor at any health facility level. There is also a child survival strategy in Ethiopia that connects essential maternal, newborn, and child health packages throughout adolescence, pregnancy, childbirth, postnatal and newborn periods, and into childhood building upon their natural interactions throughout life. Even if such strategies were applied by Ethiopian health policy the problem of newborn death was still high in the country [5, 8].

Many studies in different parts of Ethiopia showed that parity [9], anemia [10, 11], antepartum hemorrhage [12], prolonged labor [9], preterm birth [12], meconium-stained liquor [12, 13], pregnancy-induced hypertension [10], and mode of delivery [14] were significant predictors of birth asphyxia. However, information related to determinants of newborn asphyxia in Debre Berhan referral hospitals is limited. Therefore, the aim of this study was to identify determinants of birth asphyxia among newborns in Debre Berhan Referral Hospital, Debre Behan, Ethiopia. The findings will help to improve health care providers’ and women’s knowledge on birth asphyxia during labor. *This study also contributes for SGD goals 3 to reduce neonatal mortality by identifying the risk factors of birth asphyxia.*

## Methods

### Study area and period

The study was conducted in Debre Berhan referral hospital at Debre Berhan town. It is located in North Shoa Zone, Amara regional state, North East part of Ethiopia. It is 130 km far from the capital city Addis Ababa, and 695 km far from the regional city Bahir Dar. It is one of the largest public referral hospitals in the zone, providing preventive, curative and rehabilitative services to about more 3 million people in the catchment area. It also provides delivery service 24 h a day, 7 days a week by 24 midwives, 3 general practitioners and 4 gynecologists who assist about 3366 deliveries annually. In NICU ward there are 22 nurses, 3 general practitioners and 2 pediatricians. The total admission of NICU wards from 2019 report 1056 neonates 313 by birth asphyxia. The study was conducted from January 1st to March 30th, 2020.

### Study design

An institution-based unmatched case-control study design was employed to assess determinants of birth asphyxia among newborns in Debre Berhan Referral Hospital.

### Population

All newborns delivered in Debre Berhan referral hospital were the source population and all newborns delivered in the hospital during the study period were the study population. Newborns that had heart deformity and more than one malformation were excluded from the study.

### Sample size determination

The sample size was determined by Epi Info 7 version software package for the unmatched case control study. By considering, 29.4% proportion of controls exposed and odds ratio of 2 for low gestational age from a previous study conducted in Ethiopia Tigray region [12],with an assumption of, 95% confidence interval, 80% power of the study, two- to- one control to cases ratio, and 5% non-response rate. The final sample size was 276 (92 cases and 184 controls).

### Sampling procedure

A systematic random sampling technique was used to select the study participants. Every third study subjects were included for both cases and controls. On the first day of data collection the first study participant was taken by using simple random sampling technique from the registration book.

### Data collection tools and procedure

The data were collected by using interviewer administered structured questionnaire adapted from different literatures [7, 12], and document review*. The socio-demographic and antepartum characteristics of the responder were collected by using interview and intra-partum and neonate characteristics were collected from medical records of the mother by using checklist.* The questionnaire was first prepared in English and translated to local language Amhric and back translates to English to keep its consistency. The questionnaire was pre-tested in another health facilities and essential amendment were made based on the results. Data were collected by four midwives and two supervisors who have en experience of work on neonatal intensive care unit of the hospital.

### Operational definition

#### Cases

When the newborn has at least one of the following signs, not breathing, gasping, < 30 breaths per minute or *5th minute* APGAR score of < 7 [10].

#### Outcome variable

Birth asphyxia.

### Independent variable

I. *Socio demographic determinants:* age, marital status, residence, educational status, maternal occupation
II. *Ante partum determinants:* parity, pregnancy induced hypertension, anemia, ante-partum hemorrhage, ANC follow up, chronic disease
III. *Intra-partum determinants:* fetal presentation, duration of labor, amniotic fluid, type of labor, mode of delivery, labor attendant, PROM
IV. *Neonatal determinants:* Sex, birth weight, gestational age

### Data quality control

The quality of data was assured by using properly designed and validated questionnaire by those individuals who work more on the field of the study. Training was given for the data collectors and supervisors on how to collect the data for 3 days. Pre-testing was performed in another health facility and essential amendment was made on the questionnaire. The principal investigator and supervisors were made a day to day on site supervision and check the collected data for completeness, clarity and consistency.

### Data processing and analysis

The data was coded and entered to Epi-data version 3.1 and exported to SPSS Version 21 for data analysis. Descriptive statistics were performed and presented in the form of means, standard deviations, frequencies and percentages. Binary logistic regression was computed to identify determinates of birth asphyxia. Variable which have *p*-value less than 0.25 in binary logistic regression analysis were transferred to multiple logistic regression model to see the relative effect of the confounder. Model goodness of fit test was checked by using Hosmer Lemeshow goodness of fit and Multicolinearity was assessed by variance inflation factor (VIF) among variables. Adjusted odd ratios with 95% confidence interval and *P*-value less than 0.05 was used to identify determinates of birth asphyxia.

## Results

### Socio-demographic characteristics of the study participants

A total of 276 (92 cases and 184 controls) were participated in the study with a response rate of 100%. About 52.2% of cases and 42.4% controls were live in rural resident and 42.4% of the mother of the cases and 6.0% controls were can’t read and write in their educational status. More mother of cases (48.9%) than controls (19.0%) were house wife by their occupational status. The majority of mother of cases (80.4%) and controls (95.1%) were married in their marital status (Table 1).

**Table 1:** Socio demographic characteristics of the study participants in Debre Berhan referral hospital, Debre Berhan, Ethiopia, 2020

Variable	Cases (%)	Controls (%)	Total (%)
Age of the mother (year)			
< =19	8 (8.7)	5 (2.7)	13 (4.8)
20–24	30 (32.6)	38 (20.7)	68 (24.6)
25–29	28 (30.4)	74 (40.3)	102 (36.9)
30–34	14 (15.2)	33 (17.9)	47 (17)
> =35	12 (13.1)	34 (18.5)	46 (16.7)
Residence			
Urban	44 (47.8)	106 (57.6)	150 (54.3)
Rural	48 (52.2)	78 (42.4)	126 (45.7)
Marital status			
Married	74 (80.4)	175 (95.1)	249 (90.2)
Single	18 (19.6)	9 (4.9)	27 (9.8)
Maternal education			
Can’t read and write	39 (42.4)	11 (6.0)	50 (18.1)
Primary school	22 (23.9)	27 (14.7)	49 (17.8)
Secondary school	13 (14.1)	71 (38.6)	84 (30.4)
College/university	18 (19.6)	75 (40.7)	93 (33.7)
Maternal occupation			
House wife	45 (48.9)	35 (19.0)	80 (29.0)
Merchant	13 (14.1)	16 (8.7)	29 (10.5)
Farmer	8 (8.7)	11 (6.0)	19 (6.9)
Private employee	11 (12.0)	69 (37.5)	80 (29.0)
Government employee	15 (16.3)	53 (28.8)	68 (24.6)

### Ante partum related characteristics of the study participants

From the total study participants,46 (55.4%) mothers of the cases and 37 (44.6%) mothers of the controls were premipara, 39 (61.9%) mothers of the cases and 24 (38.1%) mothers of the controls were less than or equals to two ANC visits, 25 (78.1%) of the mothers of the cases and 7 (21.9%) mothers of the controls were APH during pregnancy, 21 (77.8%) mothers of the cases and 6 (22.2%) of the controls had anemia and 19 (73.1%) mothers of the cases and 7 (26.9%) of the controls were PIH during pregnancy (Table 2).

**Table 2:** Ante partum characteristics of the study participants in Debre Berhan referral hospital, Debre Berhan, Ethiopia, 2020

Variable	Cases (%)	Controls (%)	Total (%)
Parity			
1(premipara)	46 (50.0)	37 (20.1)	83 (30.1)
2–4(multipara)	29 (31.5)	129 (70.1)	158 (57.2)
> =5(grand multipara)	17 (18.5)	18 (9.8)	35 (12.7)
ANC follow up			
< = two	39 (42.3)	24 (13.0)	63 (22.8)
Three	20 (21.7)	19 (10.3)	39 (14.1)
Four and above	33 (36.0)	141 (76.7)	174 (63.1)
APH			
Yes	25 (27.2)	7 (3.8)	32 (11.6)
No	67 (72.8)	177 (96.2)	244 (88,4)
PIH			
Yes	19 (20.7)	7 (3.8)	26 (9.4)
No	73 (79.3)	177 (96.8)	250 (90.6)
Anemia			
Yes	21 (22.8)	6 (3.3)	27 (9.8)
No	71 (77.2)	178 (96.7)	249 (90.2)
Chronic disease			
Yes	13 (14.1)	14 (7.6)	27 (9.8)
No	79 (85.9)	170 (92.4)	249 (90.2)

### Intra partum related characteristics of the study participants

About 43.4% of the cases and 23.9% of the controls mother labor were attended by midwives. Majority 54.3% of the cases and 84.8% of the controls mother had spontaneous type labor and 50.0% of cases and 7.6% of controls mother delivered with stained amniotic fluid. Majority 73.9% of cases and 88.0% of controls were cephalic fetal presentation. About 14.1% of the cases and only 1.1% of controls were delivered with cored prolapsed (Table 3).

**Table 3:** Intra partum related characteristics of the study participants in Debre Berhan referral hospital, Debre Berhan, Ethiopia, 2020

Variable	Cases (%)	Controls (%)	Total (%)
Labor attendant			
Midwives	39 (43.4)	44 (23.9)	83 (30.1)
General practitioner	30 (36.6)	92 (50.0)	122 (44.2)
Gynecologist	23 (25.0)	48 (26.1)	71 (25.7)
Type of labor			
Spontaneous	50 (54.3)	156 (84.8)	206 (74.6)
Induced	42 (45.7)	28 (15.2)	70 (25.4)
Duration of labor			
Normal	24 (20.1)	151 (82.1)	175 (63.4)
Prolonged	68 (79.9)	33 (17.9)	101 (36.6)
Mode of delivery			
SVD	37 (40.2)	131 (71.2)	168 (60.9)
Instrumental	26 (28.3)	23 (12.5)	49 (17.7)
CS	29 (31.5)	30 (16.3)	59 (21.4)
Amniotic fluid			
Stained	46 (50.0)	14 (7.6)	60 (21.7)
Non stained	46 (50.0)	170 (92.4)	216 (78.3)
PROM			
Yes	39 (42.4)	25 (13.6)	64 (23.2)
No	53 (57.6)	159 (86.4)	212 (76.8)
Obstructed labor			
Yes	27 (29.3)	16 (8.7)	43 (15.6)
No	65 (70.7)	168 (91.3)	233 (84.4)
Fetal presentation			
Cephalic	68 (73.9)	162 (88.0)	230 (83.3)
Not cephalic	24 (26.1)	22 (12.0)	46 (16.7)
Cord prolapse			
Yes	13 (14.1)	2 (1.1)	15 (5.4)
No	79 (85.9)	182 (98.9)	261 (94.6)

### Newborn related characteristics of the study participants

This study revealed that 54 (58.7%) of cases and 107 (58.2%) of controls were male neonates. Twenty two (23.9%) of cases and only 6 (3.3%) of controls were preterm neonates. It was also observed that 39 (42.4%) of the cases and 34 (18.5%) of the controls were low birth weight (Table 4).

**Table 4:** Neonatal related characteristics of the study participants in Debre Berhan referral hospital, Debre Berhan, Ethiopia, 2020

Variable	Cases (%)	Controls (%)	Total (%)
Sex of new born			
Male	54 (58.7)	107 (58.2)	161 (58.3)
Female	38 (41.3)	77 (41.8)	115 (41.7)
Gestational age			
Pre-term	22 (23.9)	6 (3.3)	28 (10.1)
Term	38 (41.3)	166 (90.2)	204 (73.9)
Post-term	32 (34.8)	12 (5.5)	44 (16.0)
Birth weight			
< 2500 g	39 (42.4)	34 (18.5)	73 (26.4)
> =2500	53 (57.6)	150 (81.5)	203 (73.6)

### Determinants of birth asphyxia among newborns

*Those variables which showed p-value < 0.05 in bivariate analysis were transferred to multivariate analysis to see the effect of confounders.* In multivariate logistic regression analysis maternal education, ANC follow up, presence APH, prolonged labor, stained amniotic fluid, not cephalic fetal presentation and gestational age less than 37 weeks were identified as determinants of birth asphyxia among newborns. This study revealed that mother who can’t read and write [AOR = 4.7; 95%CI (1.2, 11.9)] were 4.7 times higher to develop birth asphyxia compared from mother who have college diploma and above. The odds of developing birth asphyxia among mothers who faced antepartum hemorrhage was 7.7 times higher compared from the counterparts [AOR = 7.7; 95%CI (1.5, 18.5)]. This study revealed that prolonged labor was the main predictor of birth asphyxia. A mother who had prolonged labor was more than 13 time higher risk compared from normal labor on the outcome of birth asphyxia [AOR = 13.5; 95%CI (2.0, 19.4)]. Preterm babies were 4.1 times higher risk than term babies to development birth asphyxia [AOR = 4.1; 95%CI (1.8, 9.2)] (Table 5).

**Table 5:** Bivariate and Multivariate logistic regression analysis on the determinants of birth asphyxia among newborns in Debre Berhan Referral hospital, Debre Berhan, Ethiopia 2020

Variable	Asphyxia status		COR (95%CI)	AOR (95%CI)
	Cases (%)	Controls (%)		
Maternal education				
Can’t read & write	39 (42.4)	11 (6.0)	14.8 (6.4, 34.4)	4.7 (1.2, 11.9)*
Primary school	22 (23.9)	27 (14.6)	3.4 (1.6, 7.3)	0.5 (0.1, 2.2)
Secondary school	13 (14.1)	71 (38.6)	0.8 (0.4, 1.7)	0.5 (0.4, 2.4)
Diploma & above	18 (19.6)	75 (40.8)	1.0	1.0
ANC follow up				
< Two	39 (42.4)	24 (13.1)	6.9 (3.7, 13.1)	4.6 (1.1, 9.5)*
Three	20 (21.7)	19 (10.3)	4.5 (2.2, 9.4)	2.4 (0.5, 11.4)
Four and above	33 (35.9)	141 (76.6)	1.0	1.0
APH				
Yes	25 (27.2)	7 (3.8)	9.4 (3.9, 22.8)	7.7 (1.5, 18.5)*
No	67 (72.8)	177 (96.2)	1.0	1.0
Type of labor				
Spontaneous	50 (54.3)	156 (84.8)	1.0	1.0
Induced	42 (45.7)	28 (15.2)	4.7 (1.1, 7.4)	6.4 (0.7, 37.2)
Duration of labor				
Normal	24 (26.1)	151 (82.1)	1.0	1.0
Prolonged	68 (73.9)	33 (13.9)	13.0 (2.4, 18.1)	13.5 (2.0, 19.4)*
Amniotic fluid				
Stained	46 (50.0)	14 (7.6)	12.1 (6.2, 24.0)	11.3 (2.7, 39.5)*
Non stained	46 (50.0)	170 (92.4)	1.0	1.0
PROM				
Yes	39 (42.4)	25 (13.6)	4.7 (2.6, 8.5)	6.1 (0.7, 8.5)
No	53 (57.6)	159 (86.4)	1.0	1.0
Obstructed labor				
Yes	27 (29.3)	16 (8.7)	4.4 (2.2, 8.6)	1.2 (0.1, 2.0)
No	65 (70.7)	168 (91.3)	1.0	1.0
Fetal presentation				
Cephalic presentation	68 (73.9)	162 (88.0)	1.0	1.0
Breech presentation	24 (26.1)	22 (12.0)	2.6 (2.0, 7.3)	4.5 (2.0, 8.4)*
Gestational age				
Pre-term (< 37)	22 (23.9)	6 (3.3)	1.4 (1.2, 6.1)	4.1 (1.8, 9.2)*
Term (37–39)	38 (41.3)	166 (90.2)	0.2 (0.1, 1.3)	0.1 (0.01, 0.5)
Post-term (> = 40)	32 (34.8)	12 (6.5)	1.0	1.0
Birth weight				
< 2500 g	39 (42.4)	34 (18.5)	3.3 (1.9, 5.7)	2.7 (0.9, 8.5)
> =2500	53 (57.6)	150 (81.5)	1.0	1.0

## Discussion

This study tried to identify the determinants of birth asphyxia among newborns in Debre Berhan referral hospital by including number of variables from different categories like socio-demographic, antepartum, intra partum and neonatal related characteristics of the newborns.

This study revealed that mothers who can’t read and write were 4.7 times higher risk to have asphyxiated newborns compared to mothers who have a college diploma and above. This finding was consistent with the study conducted in *Sweden* [15], *Nepal* [16], Pakistan [17] and Ethiopia [7, 12, 18]. This could indicate that poor literacy limits mothers from having independent decisions and good access to family resources that could restrict their health-seeking behaviour during the antepartum period and also it is an indicator of poor socio-economic status of the community and unplanned pregnancy.

Gestational age was significantly associated with birth asphyxia. Preterm (<37wks) was 4.1 times more likely asphyxiated than the term newborns. This finding was consistent with those of previous studies conducted in Pakistan [17], Nepal [16], and Ethiopia [9, 12, 19]. This may be due to the fact that preterm babies face multiple morbidities including organ system, immaturity especially lung immaturities causing respiratory failure.

Newborns with the breech presentation were 4.5 times the odds of developing birth asphyxia than the cephalic presentation. This finding was consistent with previous studies conducted in Uganda [20], Cameron [21], and Ethiopia [22]. Mal-presentation of foetus is associated with premature rupture of membrane; this premature rupture of membrane could leads to umbilical cord accidents occur with sub sequent asphyxia at birth [14]. But, the finding was inconsistent with previous studies conducted in Ethiopia [13, 23]. This difference might be due to the difference in the study population*, both of the study was conducted in metropolitan city of Ethiopia at which the maternal healthcare services were more advanced than the study area.*

Prolonged labour was significantly associated with birth asphyxia. This finding was consistent with studies conducted in *India* [24]*, Malawi* [25], Cameron [21], Pakistan [17], and Ethiopia [11, 26–28]. This could be due to primary, secondary, or tertiary dalliance. This is in fact that, when labour is prolonged there was a high probability for the foetus to become distressed which can lead to birth asphyxia [28].

Less than or equals to two ANC follow up by the mothers were 4.6 times higher odds of developing asphyxiated newborns than mothers who have more than four ANC follow up. This finding was inconsistent with the study conducted in Ethiopia [13, 23, 27]. This difference may be due to the number of health facilities included in the study and study period. This ANC visit of the pregnant mother are very important to minimize adverse pregnancy outcomes including birth asphyxia as they provide chance for evaluating the foetal wellbeing and permit management soon by improving the health and wellbeing of the mother and preventing further complications by early detection and treatment of diseases.

*In this study, induced labor doesn’t show an association with birth asphyxia when compared to Spontaneous labor. This result was congruent with the study conducted in West Shewa Zone, central Ethiopia* [22]*. But it was inconsistent with the Meta-analysis study conducted in Ethiopia* [29]*. This difference may be due to the difference in the study setting at which both this study and the central Ethiopia study were conducted in a single institution. This induction of labor may cause hyper-stimulation of the uterine constriction that could cause fetal heart rate changes. When constrictions are too fast and strong, the placenta, which helps carry oxygen-rich blood to the baby, often cannot recharge with an adequate supply of this blood for the baby* [30]*.*

In this study, we have limitations that should be noted. The study was done in single health facility therefore; it is difficult to generalize for the whole country with this small sample. This study also subjected to recalling bias of mothers when they remembered their previous history. *The study also have other limitation that variables in intrapartum events like umbilical cord status, uterine rupture, placental abruption, shoulder dystocia and major maternal haemorrhage, trauma, cardiorespiratory arrest or maternal seizures immediately preceding delivery were not addressed.*

## Conclusions

In this study, maternal education can’t read & write, antepartum hemorrhage, prolonged labour, stained amniotic fluid, breech fetal presentation, preterm birth were significantly associated with birth asphyxia. So, educating mothers *to enhance health seeking behaviors and close monitoring of the labor and fetus presentation were recommended to reduce birth asphyxia.*

## Supplementary Information

**Additional file 1.**

## Data Availability

The datasets used and/or analyzed during the current study available from the corresponding author on reasonable request.

## Abbreviations

ANC: Antenatal Care
AOR: Adjusted Odd Ratio
DDS: Dietary Diversity Score
FAO: Food and Agriculture Organization
NNP: National Nutritional Program
SD: Standard Deviation
SPSS: Statistical Package for Social Science
WHO: World Health Organization
