# Supplementary material for: Determinants of birth asphyxia among newborns in Debre Berhan referral hospital, Debre Berhan, Ethiopia: a case-control study
Source: BMC Pediatr. 2022 Mar 30;22:165. doi: 10.1186/s12887-022-03223-3 (PMC8966276; doi:10.1186/s12887-022-03223-3)
Supplement: Supplementary file 1 — Additional file 1. [file 12887_2022_3223_MOESM1_ESM.docx]

**These are the binary logistic regression analysis results**

Table 1: Binary logistic regression of socio-demographic factors of birth asphyxia among newborns in Debre Berhan referral hospital, Debre Berhan town, Ethiopia, 2020

| Variable | Cases (%) | Controls (%) | COR(95CI) | P value |
| --- | --- | --- | --- | --- |
| Age of the mother (year) |  |  |  |  |
| <=19 | 8 (8.7) | 5 (2.7) | 4.5 (1.2, 16.6) | 0.20 |
| 20-24 | 30 (32.6) | 38 (20.7) | 2.2 (1.0, 5.1) | 0.15 |
| 25-29 | 28 (30.4) | 74 (40.3) | 1.1 (0.5, 2.4) | 0.86 |
| 30-34 | 14 (15.2) | 33 (17.9) | 1.2 (0.5, 3.0) | 0.69 |
| >=35 | 12 (13.1) | 34 (18.5) | 1.0 |  |
| Residence |  |  |  |  |
| Urban | 44 (47.8) | 106 (57.6) | 1.0 |  |
| Rural | 48 (52.2) | 78 (42.4) | 1.5 (0.9, 2.5) | 0.13 |
| Marital status |  |  |  |  |
| Married | 74 (80.4) | 175 (95.1) | 1.0 |  |
| Single | 18 (19.6) | 9 (4.9) | 4.7 (2.0, 11.0) | 0.21 |
| Maternal education |  |  |  |  |
| Can’t read and write | 39 (42.4) | 11 (6.0) | 14.8 (6.4, 34.4) | 0.001 |
| Primary school | 22 (23.9) | 27 (14.7) | 3.4 (1.6, 7.3) | 0.002 |
| Secondary school | 13 (14.1) | 71(38.6) | 0.8 (0.4, 1.7) | 0.050 |
| College/university | 18 (19.6) | 75 (40.7) | 1.0 |  |
| Mothers Occupation |  |  |  |  |
| House wife | 45 (48.9) | 35 (19.0) | 4.5 (2.2, 9.4) | 0.13 |
| Merchant | 13 (14.1) | 16 (8.7) | 2.9 (1.1, 7.2) | 0.23 |
| Farmer | 8 (8.7) | 11 (6.0) | 2.6 (0.9, 7.5) | 0.39 |
| Private employee | 11 (12.0) | 69 (37.5) | 0.6 (0.2, 1.3) | 0.19 |
| Government employee | 15 (16.3) | 53 (28.8) | 1.0 |  |

Table 2: Binary logistic regression of ante partum related factors of birth asphyxia among newborns in Debre Berhan referral hospital, Debre Berhan town, Ethiopia, 2020

| Variable | Cases (%) | Controls (%) | COR(95%CI) | P value |
| --- | --- | --- | --- | --- |
| Parity |  |  |  |  |
| 1 (premipara) | 46 (50.0) | 37 (20.1) | 1.3 (0.6 - 2.9) | 0.50 |
| 2-4 (multipara) | 29 (31.5) | 129 (70.1) | 0.2 (0.1 - 0.5) | 0.10 |
| >=5 (grand multipara) | 17(18.5) | 18 (9.8) | 1.0 |  |
| ANC follow up |  |  |  |  |
| <=two | 39 (42.3) | 24 (13.0) | 6.9 (3.7, 13.1) | 0.001 |
| Three | 20 (21.7) | 19 (10.3) | 4.5 (2.2, 9.4) | 0.001 |
| Four and above | 33 (36.0) | 141 (76.7) | 1.0 |  |
| APH |  |  |  |  |
| Yes | 25 (27.2) | 7 (21.9) | 9.4 (3.9, 22.8) | 0.001 |
| No | 67 (72.8) | 177 (72.5) | 1.0 |  |
| PIH |  |  |  |  |
| Yes | 19(73.1) | 7 (3.8) | 6.6 (2.7, 16.3) | 0.95 |
| No | 73(29.2) | 177 (96.2) | 1.0 |  |
| Anemia |  |  |  |  |
| Yes | 21 (22.8) | 6 (3.3) | 8.8 (3.4, 22.7) | 0.31 |
| No | 71 (77.2) | 178 (96.7) | 1.0 |  |
| Chronic disease |  |  |  |  |
| Yes | 13(14.1) | 14 (7.6) | 2.0 (0.9, 4.5) | 0.90 |
| No | 79(85.9) | 170 (92.4) | 1.0 |  |

Table 3: Binary logistic regression of intra partum related factors of birth asphyxia among newborns in Debre Berhan referral hospital, Debre Berhan town, Ethiopia, 2020

| Variable | Cases (%) | Controls (%) | COR (95%CI) | P value |
| --- | --- | --- | --- | --- |
| Labor attendant |  |  |  |  |
| Midwifery | 39 (43.4) | 44 (23.9) | 1.7 (0.9, 3.3) | 0.12 |
| General practitioner | 30 (36.6) | 92 (50.0) | 3.4 (0.1, 8.4) | 0.99 |
| Gynecologist | 23 (25.0) | 48 (26.1) | 1.0 |  |
| Type of labor |  |  |  |  |
| Spontaneous | 50 (54.3) | 156 (75.7) | 1.0 |  |
| Induced | 42 (45.7) | 28 (40) | 4.7 (1.1, 7.4) | 0.001 |
| Duration of labor |  |  |  |  |
| Normal | 24 (20.1) | 151 (82.1) | 1.0 |  |
| Prolonged | 68 (79.9) | 33 (17.9) | 13.1 (2.4, 18.1) | 0.001 |
| Mode of delivery |  |  |  |  |
| SVD | 37 (40.2) | 131 (71.2) | 1.0 |  |
| Instrumental | 26 (28.3) | 23 (12.5) | 0.3 (0.2, 0.6) | 0.31 |
| CS | 29 (31.5) | 30 (16.3) | 1.2 (0.6, 2.5) | 0.69 |
| Amniotic Fluid |  |  |  |  |
| Stained | 46 (50.0) | 14 (7.6) | 12.1 (6.2, 24.0) | 0.001 |
| Non stained | 46 (50.0) | 170 (92.4) | 1.0 |  |
| PROM |  |  |  |  |
| Yes | 39 (42.4) | 25 (13.6) | 4.7 (2.6, 8.5) | 0.001 |
| No | 53 (57.6) | 159 (86.4) | 1.0 |  |
| Obstructed labor |  |  |  |  |
| Yes | 27 (29.3) | 16 (8.7) | 4.4 (2.2, 8.6) | 0.001 |
| No | 65 (70.7) | 168 (91.3) | 1.0 |  |
| Fetal Presentation |  |  |  |  |
| Cephalic | 68 (73.9) | 162 (88.0) | 1.0 |  |
| Not cephalic | 24 (26.1) | 22 (12.0) | 2.6 (2.0, 7.0) | 0.01 |
| Cord prolapse |  |  |  |  |
| Yes | 13 (14.1) | 2 (1.1) | 15.0 (3.3, 67.9) | 0.21 |
| No | 79 (85.9) | 182 (98.9) | 1.0 |  |

Table 4: Binary logistic regression of neonatal related factors of birth asphyxia among newborns in Debre Berhan referral hospital, Debre Berhan town, Ethiopia, 2020

| Variable | Cases (%) | Controls (%) | COR (95%CI) | P value |
| --- | --- | --- | --- | --- |
| Sex of new born |  |  |  |  |
| Male | 54 (58.7) | 107 (58.2) | 1.1 (0.6, 1.7) | 0.93 |
| Female | 38 (41.3) | 77 (41.8) | 1.0 |  |
| Gestational age |  |  |  |  |
| Pre-term | 22 (23.9) | 6 (3.3) | 1.4 (1.2, 6.1) | 0.001 |
| Term | 38 (41.3) | 166 (90.2) | 0.2 (0.1, 1.3) | 0.19 |
| Post-term | 32 (34.8) | 12 (5.5) | 1.0 |  |
| Birth weight |  |  |  |  |
| <2500gm | 39 (42.4) | 34 (18.5) | 3.3 (1.9, 5.7) | 0.001 |
| >=2500 | 53 (57.6) | 150 (81.5) | 1.0 |  |
